# A Comparative Study of Microleakage on Dental Surfaces Bonded with Three Self-Etch Adhesive Systems Treated with the Er:YAG Laser and Bur

**DOI:** 10.1155/2016/2509757

**Published:** 2016-06-22

**Authors:** Youssef Sanhadji El Haddar, Sibel Cetik, Babak Bahrami, Ramin Atash

**Affiliations:** ^1^Department of Stomatology and Dentistry, Erasmus Hospital, Université Libre de Bruxelles, 1070 Brussels, Belgium; ^2^Laboratory of Physiology and Pharmaceutics, Faculty of Medicine, Université Libre de Bruxelles, 1070 Brussels, Belgium

## Abstract

*Aim*. This study sought to compare the microleakage of three adhesive systems in the context of Erbium-YAG laser and diamond bur cavity procedures. Cavities were restored with composite resin.* Materials and Methods*. Standardized Class V cavities were performed in 72 extracted human teeth by means of diamond burs or Er-YAG laser. The samples were randomly divided into six groups of 12, testing three adhesive systems (Clearfil s^3^ Bond Plus, Xeno® Select, and Futurabond U) for each method used. Cavities were restored with composite resin before thermocycling (methylene blue 2%, 24 h). The slices were prepared using a microtome. Optical microscope photography was employed to measure the penetration.* Results*. No statistically significant differences in microleakage were found in the use of bur or laser, nor between adhesive systems. Only statistically significant values were observed comparing enamel with cervical walls (*p* < 0.001).* Conclusion*. It can be concluded that the Er:YAG laser is as efficient as diamond bur concerning microleakage values in adhesive restoration procedures, thus constituting an alternative tool for tooth preparation.

## 1. Introduction

The Er-YAG is currently the best adapted laser for dental applications, due to its wavelength coinciding with the water and hydroxyapatite peaks of absorption, thus conferring the ability to be very well absorbed in the dental tissues it targets, while also causing only limited penetration [1]. The transmitted energy has a thermomechanical effect on the water contained in the enamel and dentin.

As there is more water contained in decayed dentinal tissue than in healthy dentinal tissue, the treatment is more efficient on the decayed dentin, enabling selective tissue ablation. These observations are in line with the current dentistry approach of restorative dentistry, which protects the dental structure integrity by using the least invasive means possible. In this respect, Er:YAG lasers represent an ideal tool for modern dentistry. However, several parameters must still be studied, particularly in terms of the efficiency of the adhesive systems used on the surfaces undergoing these techniques.

The results presented in the literature on this matter are, in fact, highly divergent. Some studies have focused on the morphostructural analysis of the dental tissue following laser ablation as demonstrating an architecture in favor of bonding [2], whereas others have argued the contrary [3, 4].

We focused on one of the principal determining parameters of bonding quality: microleakage. Studies on this subject also present numerous contradictions. Some authors have reported unacceptably high microleakage values [5–8] with lasers, though their results are questionable, due to the use of excessive energy values (>300 mJ) during treatment. In contrast, other authors have reported the lack of significant differences between burs and lasers [9–14], whereas others have asserted that better waterproof values can be obtained with lasers compared to burs [15, 16].

Our current study thus sought to help clarify this question of microleakage from adhesive systems used on dental surfaces treated with Erbium-YAG laser.

## 2. Materials and Methods

### 2.1. Sample Selection

We included 72 extracted human wisdom teeth, all without crown changes, in this* in vitro* study. Osseous and gingival tissues as well as any residual calculus were resected by means of gouge plier and ultrasound. They were then cleaned and preserved in a physiological salt solution (0.9% NaCl) at room temperature prior to the experimental phase, in accordance with the recommendations of the International Organization for Standardization (ISO) [17].

### 2.2. Cavity Preparations

All samples were prepared by creating class V cavities using either a diamond bur or laser device.

The countersunk cavities were created by means of a diamond bur (Komet; 012 flat-end chuck cylinder) at high speed with abundant water spraying. These were performed in every 5th cavity in order to maintain an optimal ablation capacity and limit heating.

The laser-created cavities were prepared by means of an Er:YAG device (Fidelis Plus III, Fotona, Slovenia) with an R14 handpiece, on an articulated arm. The device parameters were chosen according to the manufacturer's recommendations: 300 mJ/pulses in 30 Hz, with a power of 9 Watts [18] for the enamel; 200 mJ/pulse in 20 Hz with a power of 4 Watts 20 for the dentine; duration of impulse: 100 *μ*s (very short pulse) [19].

The system includes application of a cooling spray and enabled us to perform ablation without causing thermal damage to surrounding tissues. The radiation was delivered perpendicularly to the dental surfaces, maintaining a distance of approximately 6 mm during the operation.

Each cavity was created half in the enamel and half in cement. The chosen dimensions were as follows: 1.5 mm deep, 4 mm in length in the mesiodistal direction, and 2 mm in height. By using a periodontal probe (PCP UNC 15, Hu-Friedy, Chicago) during the procedures, we were able to scrupulously adhere to the previously defined dimensions.

### 2.3. Composite Resin Bonding

All the samples prepared in the laser (*n* = 36) and bur (*n* = 36) categories were divided into three groups (*n* = 12 in each) in order to test the microleakage of three different self-etching adhesive systems (Clearfil s^3^ Bond Plus, Kuraray, Japan/Xeno Select, Dentsply, United States/Futurabond U, VOCO, Germany). Each of these systems was applied following the manufacturer's instructions. All the cavities were then restored with a composite resin (Filtek*™* Supreme, 3M, United States), polymerized for 20 sec with an LED lamp (Elipar*™* S10, 3M, United States, maximum intensity = 1200 MW/cm^2^), and polished by means of abrasive discs of decreasing size (Sof-Lex*™*, 3M, United States).

The roots were then partially sectioned, with a color code attributed to each of the six groups in order to enable differentiation of the samples after 24 h thermocycling. The apexes were sealed with wax (Cavex Set Up Regular, Cavex, Netherlands). A colored varnish corresponding to each group was coated on the teeth in order to cover them completely, preserving the restorations and 1 mm of dental tissue around them. This was to prevent any excessive infiltration of the coloring agent, which would have made the results unfit for exact interpretation (see Table 1).

The filling then underwent an ageing process, boosted by thermocycling (2500 cycles of dumping in a bath of 0°C then of 50°C at 30 sec per bath), and then plunged into 2% methylene blue for 24 hours. Those samples were then abundantly rinsed to eliminate any excess of coloring agent.

### 2.4. Cylinder Shaping

The objective of this step was to block the cavities in a suitable position for performing histological cuts. For that purpose, the teeth had to be fixed to zinc-plated blind with one eye nut (diameter M6) to be correctly positioned to be screwed to the microtome for making the cuts. The shaping process was as follows:

(i) Sanding the nuts to increase the retention of the envelopment resin.
(ii) Cutting 20 mL plastic irrigation syringes of 20 mL (Terumo®, Japan) with a manual saw to serve as a mold.
(iii) Sample orientation to obtain a cut of the cavity in the sagittal plan and adhesion of these to the nut with wax (Cavex Set Up Regular, Cavex, Netherlands).
(iv) Positioning of the tooth/nut system in the center of the mold on a Vaseline-coated glass plate.
(v) Coating with some transparent polymethyl methacrylate resin (Orthocryl, Dentaurum, Germany).
(vi) Resin cylinder polymerization in a pressure cooker (*T*° ~ 50°C; *p*° = 2 bars) for 15 min.

### 2.5. Histological Slice Procedure

After demolding, cylinders were fixed to a microtome (Leitz on 1600, Solms, Germany) through the nut. First, cuts were made at the cavity level in order to set up the section plane within the zone of interest. After securing one side with a small amount of cyanoacrylate glue, a second cut was made to obtain 700 *μ*m histological slices.

Slices were then submitted to microscopic analysis to assess the degree of penetration of the coloring agent in the enamel and cementum walls. A score of 0–3 was attributed as shown in Table 2.

**Figure 1:**
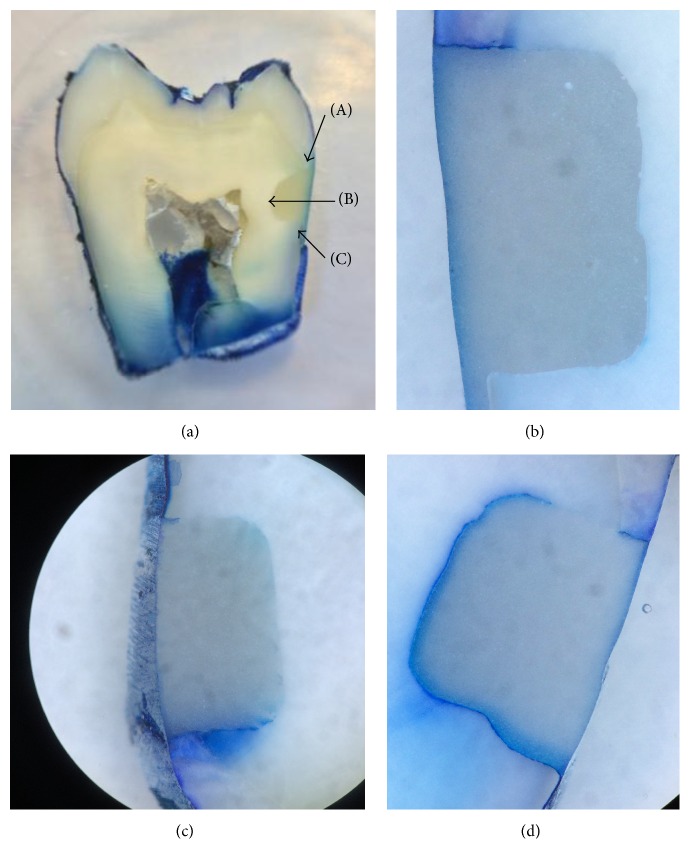
Illustration of the scoring system (a) macroscopic view; score 0: no infiltration. “A” represents the enamel wall, “B” the pulpal wall, and “C” the cement wall. (b) microscopic view; score 1: infiltration (here: enamel infiltration) inferior to the half of the wall length. (c) microscopic view; score 2: infiltration (here: cement infiltration) superior to the half of the wall length, without penetrating the pulpal wall. (d) microscopic view; score 3: infiltration (here: cement infiltration) with pulpal infiltration.

Figure 1 provides a visual description of the scoring.

In order to guarantee assessment objectivity, a double-blind analysis was performed, in which every blade saw was assigned a random number (1–72) determined by a randomization algorithm. All samples could, thus, be studied without knowing the group to which they belonged. Three examiners then analyzed all samples and scored them according to the above-mentioned methodology.

In the cases where a difference between investigators' observations was noted, the observations were discussed until a consensus was reached. The results were then listed in a contingency table. The collected information was rearranged to allow for comparison of waterproof quality between

- enamel and cement walls,
- bur curettage and laser,
- the various adhesive systems used.

These results were submitted to statistical analysis, using chi-squared test.

## 3. Results

Tables 3 [Table tab4]–5 display the results of the microleakage scores reported in this study. These were submitted to chi-squared tests in order to estimate whether there was a difference in waterproof quality between the enamel and cement, between curettage with laser and with a bur, and finally between the different adhesive systems.

(I) A highly significant difference (*p* = 0.001) was found between the infiltration of coloring agent in the enamel and the dentine, all groups considered.
(II) A statistically significant difference (0.01 < *p* < 0.05) was found concerning the infiltration of the coloring agent in the enamel between the bur and laser techniques, regardless of adhesive system type.
(III) Concerning the cement, no statistically significant difference was noted between the laser and bur (*p* > 0.05).
(IV) There was no statistically significant difference between the various adhesive systems used (*p* > 0.05).

## 4. Discussion

In this study, samples from all groups exhibited less microleakage at the cervical wall, in line with reports published by several authors [8, 20, 21]. This can be accounted for by the fact that adhesion to the dentin is more technical and dependent upon substract bonding to the enamel. On the other hand, careful observation of the enamel walls revealed infiltration limited to the enamel-dentin junction in most samples. This observation aligns with those made by Setien et al., who also observed infiltration in the enamel in samples without previous etching [22]. Ceballos et al. obtained similar results and described infiltration of 90.7% of the enamel when only lasers were used [7].

The same authors observed infiltration of coloring agent in all groups, which reached the pulp wall in most cases, an observation also made during our study. Concerning the difference of microleakage between the laser curettage method and that using a bur, no statistically significant difference was observed, in line with numerous conclusions found in literature [7, 9, 23, 24].

However, several authors assert that less microleakage occurs using lasers if preliminary etching is performed [5, 6, 25–27]. In contrast, other recent authors propose better adhesion and bonding strength with the laser [28–30] and those are considered even better when the enamel is etched [31]. The results of our study demonstrated that, at the enamel level, the coloring agent penetrated less in the laser group samples. One explanation for this was that we obtained dental surfaces without fragments, with “smear layer” or oil forming a microretentive surface during the laser procedure, as shown in some studies or environmental scanning electron microscope (ESEM) analysis [32, 33]. Our results should nevertheless be interpreted with caution, being, on the one hand, not highly significant and, on the other hand, referring to surfaces curetted with the bur that underwent no acid processing. Finally, none of the tested adhesive systems demonstrated a superior performance to any other, irrespective of sample group. Literature reports that Clearfil s^3^ Bond presented the highest microtensile bond strength to dentin in both laser-irradiated and bur-cut cavity preparation methods in studies involving self-etch adhesive systems [34].

## 5. Conclusion

Based on our results, and within the limits of this study, we conclude that, in terms of microleakage, there is no difference between the bur technique of cavity preparation and that using an Er:YAG laser. The laser can be used as an alternative to the bur for the cavity preparation. Furthermore, none of the tested adhesive systems proved superior to any other.

## Figures and Tables

**Table 1:** Color code chosen for the different sample groups.

Curettage method	Used adhesive system	Color of the varnish
Laser Er:YAG	Clearfil s^3^ Bond Plus	Blue
	Xeno Select	Yellow
	Futurabond U	Red

Fraise	Clearfil s^3^ Bond Plus	Green
	Xeno Select	Pink
	Futurabond U	Orange

**Table 2:** Criteria used to score the infiltration.

Score	Location of the infiltration
0	No infiltration
1	Half wall
2	Infiltration from half the wall to the whole wall without penetrating the pulp wall
3	Pulp wall

**(a):** 

	Score 0	Score 1	Score 2	Score 3
Clearfil s^3^ Bond Plus	3	8	0	1
Xeno Select	2	10	0	0
Futurabond U	6	4	1	1

**(b):** 

	Score 0	Score 1	Score 2	Score 3
Clearfil s^3^ Bond Plus	1	9	2	0
Xeno Select	0	10	1	1
Futurabond U	1	9	1	1

**(a):** 

	Score 0	Score 1	Score 2	Score 3
Clearfil s^3^ Bond Plus	1	0	0	11
Xeno Select	0	3	1	8
Futurabond U	1	1	0	10

**(b):** 

	Score 0	Score 1	Score 2	Score 3
Clearfil s^3^ Bond Plus	1	2	1	8
Xeno Select	1	1	1	9
Futurabond U	2	0	0	10

**(a):** 

	Score 0	Score 1	Score 2	Score 3
A	13	50	5	4
B	6	7	3	56

**(b):** 

	Score 0	Score 1	Score 2	Score 3
A	11	22	1	2
B	2	28	4	2

	Score 0	Score 1	Score 2	Score 3

C	2	4	1	29
D	4	3	2	27

**(c):** 

	Score 0	Score 1	Score 2	Score 3
Clearfil s^3^ Bond Plus	6	19	3	20
Xeno Select	3	24	3	18
Futurabond U	10	14	2	22
